# 

**Published:** 1917-12-29

**Authors:** 


RATES OF SUBSCRIPTION: Twelve months, 6/6; Six months, 4/-; three months, 2/-, post free to any address in the United Kingdom. Abroad, Twelve months, 8/8; Six months, 5/-; Three months, 2/6, post free. THE HOSPITAL "Index" to Volume LXII., April 1917 to September 1917, is now ready, price 2½d. per copy, post free. Address The Manager, THE HOSPITAL, "The Hospital" Building, 28/29 Southampton Street, Strand, London, W.C. 2.

### Male Red Cross Workers.

The total number of men of military age employed by the British Red Cross Society in France is approximately 1,900. A proportion of these men are being withdrawn for service in the armed forces, on the principle that men fit for general service and men of category B 1 under thirty years of age shall be released, subject to the right of appeal by the Society to the Field-Marshal Commanding-in-Chief in France in very special cases of indispensability. The Society has about 200 men of military age employed in other theatres of the war, and, excluding Tribunal cases, 250 at home who, with two exceptions, are not fit for general service. In addition there are a certain number who have been granted exemption by Tribunals, over whom the National Service Ministry and the War Office have no control.

### Matrons' and Sisters' Appointments.

Ear and Throat Hospital, Edmund Street, Birmingham.—Miss Annie Strachan has been appointed matron. She was trained at St. Helen's Hospital, and has been assistant matron at the County Hospital, Lincoln, and matron at Wallasey Cottage Hospital.

Aberdare and District General Hospital.—Miss C. Vasey and Miss Marion Pattie Thomas have been appointed ward sisters. Miss Vasey was trained at the Anlaby Road Infirmary, Hull, where she has since been ward sister. Miss Thomas was trained at Pontypool General Hospital, and has been staff nurse at Pontypridd General Hospital.

City of Westminster Union Infirmary.—Miss Ethel Elizabeth Harris has been appointed sister. She was trained at the Union Hospital, Cardiff, where she was later sister.

St. John War Auxiliary Hospital, Hastings.—Miss Olive Hannah Smith has been appointed night sister. She was trained at the City of Westminster Infirmary, later being staff nurse and sister there. She has also been ward sister at the Children's Hospital, Cleveland Street, W., and theatre and out-patients' sister at Seaford Military Hospital (Surrey Convalescent Home).

Upperton Red Cross Hospital, Eastbourne.—Miss Edith Holdstock has been appointed night sister. She was trained at Greenwich Infirmary, and has been sister at Lewisham Military Hospital.

V.A.D. Hospital, Bournemouth.—Miss M. E. Smith has been appointed sister. She was trained at the Coventry and Warwickshire Hospital, later being theatre sister there. She has been staff nurse and had theatre charge at the New Hospital for Women, London, sister at the Homœopathic Hospital, Birmingham, and at the Women's Hospital, Rochdale, charge sister at the Beaufort War Hospital, Fishponds, Bristol, and night superintendent at the District Hospital, Walsall.

V.A.D. Hospital, Winslow, Bucks.—Miss Bruce has been appointed sister-in-charge. She was trained at the Radcliffe Infirmary, Oxford, and has held posts at the V.A.D. No. 5 Hospital, Exeter, and at Wray House Auxiliary Hospital.

Ward Auxiliary Military Hospital, Reigate.—Mrs. L. M. Evans has been appointed sister. She was trained at Cheltenham General Hospital, later being ward sister there, and has been ward sister at the Military Hospital, Whitchurch, Cardiff.

East London Hospital for Children, Shadwell.—Miss A. M. Coulton has been appointed lady superintendent. She was trained at Guy's Hospital and the Children's Infirmary, Liverpool, where she was later sister. Miss Coulton has been surgical night sister, out-patient sister, and assistant matron at Guy's Hospital.

**NOTE.—Particulars of Appointments for insertion in this column will be welcomed.**

### The Book Market.

LIST OF NEW BOOKS RECEIVED FROM THE FOLLOWING PUBLISHERS :—

T. Fisher Unwin, Ltd. : "The Road to a Healthy Old Age." By T. Rodley Scott, M.R.C.S., L.R.C.P. 4s. 6d. net.

Methuen and Co., Ltd. : "The Baby." By Arthur Saunders. 1s. 3d. net.

J. M. Dent and Sons, Ltd. : "A Complete System of Nursing." By A. Millicent Ashdown. 10s. 6d. net.

Macmillan and Co., Ltd. : "Lord Lister." By Sir Rickman John Godlee, Bart. 18s. net.

H. K. Lewis and Co., Ltd : "The Theory and Practice of Massage." By B. Goodall Copestake. 8s. 6d. net. "Venereal Diseases and their Prevention." By S. Winkfield Williams. 6d. net.

### Papers, Pamphlets, etc.

Hongkong. Medical and Sanitary Reports for the Year 1916.

University of Durham, College of Medicine, Newcastle-on-Tyne. Calendar for the Year 1917-1918.

Burns and Oates : "Dublin Review." October 1917. 5s. 6d. net.

